# Serum autotaxin is a prognostic indicator of liver-related events in patients with non-alcoholic fatty liver disease

**DOI:** 10.1038/s43856-024-00499-7

**Published:** 2024-04-16

**Authors:** Takanobu Iwadare, Takefumi Kimura, Taiki Okumura, Shun-ichi Wakabayashi, Taro Nakajima, Shohei Kondo, Hiroyuki Kobayashi, Yuki Yamashita, Ayumi Sugiura, Naoyuki Fujimori, Tomoo Yamazaki, Hideo Kunimoto, Satoshi Shimamoto, Koji Igarashi, Satoru Joshita, Naoki Tanaka, Takeji Umemura

**Affiliations:** 1 Department of Medicine, Division of Gastroenterology and Hepatology, Shinshu University School of Medicine, Matsumoto, Japan; 2 Consultation Center for Liver Diseases, Shinshu University Hospital, Matsumoto, Japan; 3 Department of Gastroenterology, Maruko Central Hospital, Ueda, Japan; 4 Department of Hepatology, Shinshu Ueda Medical Center, Ueda, Japan; 5 Department of Medicine, University of California San Diego, La Jolla, CA USA; 6 Department of Hepatology, Nagano Municipal Hospital, Nagano, Japan; 7 Bioscience Division, TOSOH Corporation, Ayase, Kanagawa Japan; 8 Department of Global Medical Research Promotion, Shinshu University Graduate School of Medicine, Matsumoto, Japan; 9 International Relations Office, Shinshu University School of Medicine, Matsumoto, Japan; 10 Research Center for Social Systems, Shinshu University, Matsumoto, Japan

**Keywords:** Gastroenterology, Prognostic markers, Endocrine system and metabolic diseases

## Abstract

**Background:**

Circulating autotaxin (ATX) levels have been reported to correlate with liver inflammation activity and liver fibrosis severity in patients with non-alcoholic fatty liver disease (NAFLD). The objective of this study is to investigate whether serum ATX could predict liver-related events (LRE) in NAFLD patients.

**Methods:**

This retrospective investigation includes 309 biopsy-proven NAFLD patients registered at Shinshu University Hospital. All patients are followed for at least 1 year, during which time the prevalence of LRE, including newly developing hepatocellular carcinoma, hepatic encephalopathy, ascites, and esophagogastric varices, is investigated in relation to ATX levels at the time of liver biopsy.

**Results:**

During the median follow-up period of 7.0 years, LRE are observed in 20 patients (6.5%). The area under the receiver operating characteristic curve and cut-off value of serum ATX for predicting LRE are 0.81 and 1.227 mg/l, respectively. Multivariate Cox proportional hazards models for LRE determine ATX and advanced fibrosis as independently associated factors. Furthermore, in a competing risk analysis that considered non-liver-related death as a competing event, ATX (HR 2.29, 95% CI 1.22–4.30, *p* = 0.010) is identified as an independent factor associated with LRE, along with advanced fibrosis (HR 8.01, 95% CI 2.10–30.60, *p* = 0.002). The predictive utility of ATX for LRE is validated in an independent cohort.

**Conclusions:**

Serum ATX may serve as a predictive marker for LRE in patients with NAFLD.

## Plain language summary

In non-alcoholic fatty liver disease (NAFLD), fat accumulates and can cause damage within the liver. The disease is becoming increasingly common worldwide. It is therefore important to identify individuals with NAFLD who are at higher risk of developing severe liver complications. In this study, we found that NAFLD patients with elevated levels of a substance called autotaxin (ATX) in their blood were more prone to liver-related issues. Thus, it is crucial for doctors to give special attention to NAFLD patients exhibiting high ATX levels. Through close ATX monitoring and appropriate treatment, doctors can potentially enhance their health outcomes and prevent the onset of more severe liver complications.

## Abstract (web summary)

Iwadare et al. investigate the value of serum autotaxin levels in predicting liver-related events within a retrospective cohort of 309 biopsy-proven NAFLD patients. Multivariate Cox proportional hazards models identify autotaxin levels and advanced fibrosis as independent predictive factors.

## Introduction

As a hepatic manifestation of metabolic syndrome, non-alcoholic fatty liver disease (NAFLD) is closely linked to obesity, hypertension (HT), diabetes mellitus (DM), and dyslipidemia (DL)^1^, and is increasing worldwide^2^. NAFLD is categorized as non-alcoholic fatty liver and non-alcoholic steatohepatitis, the latter of which raises the risk of cirrhosis, liver failure, and hepatocellular carcinoma (HCC)^3^. In patients with NAFLD, liver fibrosis is considered the best indicator of long-term clinical prognosis^4,5^, and so the establishment of accurate, minimally invasive, and safe markers of liver fibrosis are desired^6–8^. Criticism has been directed at the terms non-alcoholic and fatty due to perceived flaws, lack of precision, and the potential for stigmatization. Consequently, a more encompassing term, steatotic liver disease (SLD), has been adopted to cover a spectrum of conditions causing steatosis, incorporating metabolic dysfunction and alcoholic liver disease^9^. In the new concept of SLD, NAFLD has been moved to the term “metabolic dysfunction-associated fatty liver disease” (MASLD)^9^.

Autotaxin (ATX) was originally discovered in human melanoma cell cultures^10^. The protein is encoded by the ectonucleotide pyrophosphatase/phosphodiesterase family member 2 gene, which catalyzes the hydrolysis of lysophosphatidylcholine to lysophosphatidic acid (LPA) and functions as a phospholipase^11^. We have shown that serum ATX levels correlate with liver inflammation activity and fibrosis severity in viral hepatitis, primary biliary cholangitis, and NAFLD^12–15^. Since 2018, serum ATX measurement has been covered by Japanese national health insurance for patients with chronic hepatitis and liver cirrhosis, and its clinical application is already established. However, to the best of knowledge, no studies have evaluated the significance of ATX as a predictor of NAFLD outcome. We therefore investigated whether circulating ATX levels reflected the risk of liver-related events (LRE) in NAFLD patients. In this study, a multivariate Cox proportional hazards model identified serum ATX levels as an independent predictor of LRE, along with advanced fibrosis.

## Methods

### Patients and clinical examinations

**Fig. 1:**
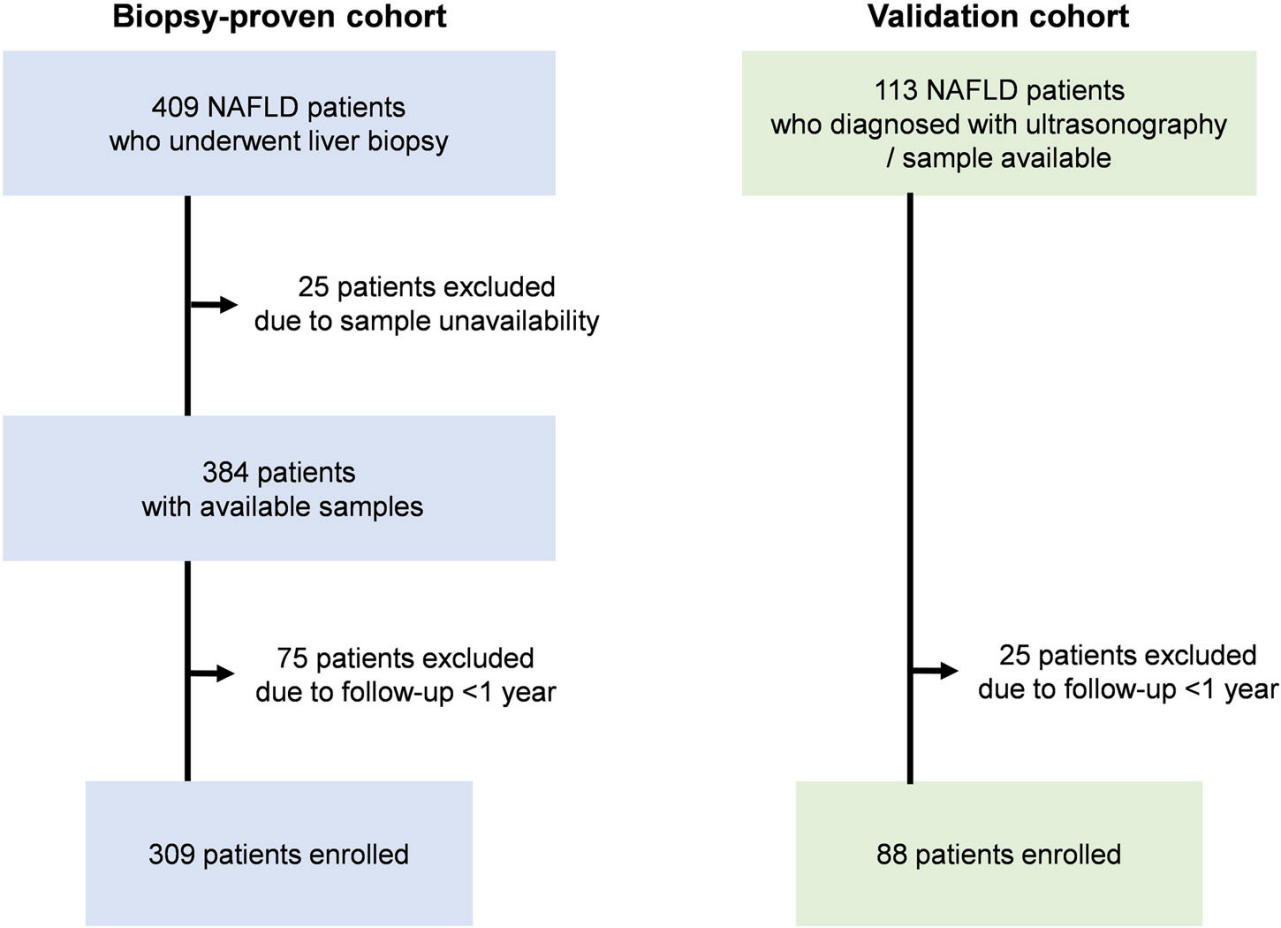
Patient inclusion flowchart for this study. NAFLD non-alcoholic fatty liver disease.

This retrospective study, including all cohorts, was approved by the Committee for Medical Ethics of Shinshu University School of Medicine (ID number: 4285) and performed in accordance with the Helsinki declaration of 1975, 1983 revision. Written informed consent was obtained from the patients who participated in this study. We firstly enrolled 409 biopsy-proven Japanese NAFLD patients who were admitted to Shinshu University Hospital (Matsumoto, Japan) between January 1998 and September 2021. NAFLD was suspected based on the following criteria: (1) the presence of hepatorenal contrast and increased hepatic echogenicity on abdominal ultrasonography; (2) an average daily consumption of <20 g of ethanol; and (3) the absence of other causes of liver dysfunction, such as viral hepatitis, drug-induced liver injury, autoimmune liver disease, primary sclerosing cholangitis, Wilson’s disease, hereditary hemochromatosis, and citrin deficiency^16^. The diagnosis of NAFLD was confirmed using the histological findings of biopsied specimens. Of the liver biopsy-diagnosed 409 NAFLD patients, 384 had serum samples available at the time of liver biopsy, among which 309 were followed for at least 1 year and included in the study (biopsy-proven cohort, Fig. 1 and Supplementary Data 1). As a validation cohort, we enrolled 88 patients diagnosed with NAFLD using ultrasonography at Shinshu University Hospital between February 2019 and November 2022. In the validation cohort, NAFLD was diagnosed based on the following criteria: (1) the presence of hepatorenal contrast and increased hepatic echogenicity on abdominal ultrasonography; (2) an average daily consumption of <20 g of ethanol; and (3) the absence of other causes of liver dysfunction, such as viral hepatitis, drug-induced liver injury, autoimmune liver disease, primary sclerosing cholangitis, Wilson’s disease, hereditary hemochromatosis, and citrin deficiency^16^. This validation cohort was followed for at least 1 year. Inclusion criteria for these patients involved the availability of serum samples for ATX measurement. Both cohorts did not include patients who developed LRE prior to NAFLD diagnosis.

Patients were considered to have HT if their systolic/diastolic pressure was >140/90 mmHg or if they were taking anti-hypertensive drugs^17^. Patients were judged as having DL if their fasting serum levels of total cholesterol (TC), low density lipoprotein cholesterol (LDL-C) or triglycerides were ≥220 mg/dl, ≥140 mg/dl, or ≥150 mg/dl, respectively, or if they were taking lipid-lowering drugs^18^. Patients were considered to have DM with a fasting glucose level of ≥126 mg/dl or hemoglobin A1c of ≥6.5%, or if they were taking insulin or oral hypoglycemic agents^19^. Body weight and height were measured before liver biopsy in an overnight fasting state. All laboratory data were obtained in an overnight fasting state on the day of liver biopsy. Homeostasis model assessment for insulin resistance (HOMA‐IR) was calculated according to the following formula: HOMA‐IR = (fasting blood glucose [mg/dl] × fasting insulin [μU/ml])/405^20^.

ATX was measured using serum samples at the time of NAFLD diagnosis. The serum ATX concentrations were determined using a 2-site immunoenzymetric assay with the TOSOH AIA system (TOSOH, Tokyo, Japan)^21^. The antibody used in this study consists of a solid-phase antibody produced by a rat anti-human ATX monoclonal antibody-producing cell clone, designated as R10.23, and a labeled antibody using the clone designated as R10.21^22^.

### Histological examinations

Liver specimens of at least 1.5 cm in length were obtained from segments 5 or 8 using a 14-gauge needle as described previously and immediately fixed in 10% neutral formalin^8^. Sections of 4 μm in thickness were cut and stained using the hematoxylin and eosin and Azan-Mallory methods. The histological activity of NAFLD was assessed by an independent expert pathologist in a blinded manner according to the established NAFLD scoring system^23^. Steatosis was graded as 0 to 3 based on the rate of steatotic hepatocytes (<5%, 5–33%, >33–66%, and >66%, respectively). Lobular inflammation was graded as 0 to 3 based on the overall assessment of all inflammatory foci (no foci, <2 foci/200× field, 2–4 foci/200× field, and >4 foci/200× field, respectively). Ballooning grade was scored as 0–2 by the frequency of ballooned hepatocytes (none, few, and many, respectively). Fibrosis stage was scored as follows: F0, none; F1, perisinusoidal or periportal; F2, perisinusoidal and portal/periportal; F3, bridging fibrosis; and F4, cirrhosis. A NAS score of 5 or greater was stipulated as the threshold for identifying cases of NASH^24^. F3 and F4 were delineated as instances of advanced fibrosis^25^.

### Patient follow-up

Patients were regularly monitored at intervals of at least 12 months using ultrasonography or computed tomography. Additionally, serum alpha-fetoprotein (AFP) levels were measured during these assessments. Cirrhotic patients underwent more frequent evaluations, with assessments scheduled at least every 6 months^26,27^. The radiological diagnosis of HCC was based on the American Association for the Study of Liver Diseases practice guidelines on the management of HCC as either: (1) the presence of a hepatic lesion >2 cm in diameter with typical vascular pattern for HCC on one dynamic imaging technique or AFP > 200 ng/ml; or (2) the presence of a lesion 1–2 cm in diameter with typical vascular pattern for HCC on two dynamic imaging techniques^28^.

LRE were defined as the development of HCC, hepatic encephalopathy of grade II or higher, poorly controlled ascites requiring hospitalization, and esophagogastric varices requiring endoscopic ligation, sclerotherapy, and balloon-occluded retrograde transvenous obliteration, including varices rupture. Follow-up time was defined as the number of years from NAFLD diagnosis to event diagnosis or from NAFLD diagnosis to the last follow-up visit when protocol surveillance confirmed no event.

### Statistical analysis

Clinical data were expressed as the number (percentage) or as the median (interquartile range [IQR]). Statistical analyses were performed using StatFlex Ver. 7.0 (Artech Co., Ltd., Osaka, Japan) and R software ver. 4.3.0. The Mann–Whitney *U* test and Chi-square test were employed for comparisons between the study groups. Diagnostic accuracy was evaluated using the area under the receiver operating characteristic curve (AUROC). The Youden index identified cut-off values, with the nearest clinically applicable value to the cut-off considered the optimal threshold for clinical convenience. The Kaplan–Meier method and log-rank testing were used to estimate disease progression. The Cox proportional hazards model was adopted to assess univariate and multivariable covariates for LRE. All statistical tests were two-tailed and evaluated at the 0.05 level of significance.

### Reporting summary

Further information on research design is available in the Nature Portfolio Reporting Summary linked to this article.

## Results

### Baseline characteristics in the biopsy-proven cohort

The clinicopathological features of the 309 patients with NAFLD in the biopsy-proven cohort who were monitored for more than 1 year are presented in Table 1. Median age at the time of biopsy was 56 years, and 135 patients (44%) were male. The elevated complication rates of DM (39%), HT (42%), and DL (63%) were typical for a NAFLD population. The median values for body mass index (BMI), aspartate aminotransferase (AST), alanine aminotransferase (ALT), and HOMA‐IR were 26.6 kg/m^2^, 49 U/l, 71 U/l, and 3.5, respectively. Median serum ATX was 0.87 mg/l. The histopathological classification by steatosis grade 1/2/3 was 96/138/75 patients, respectively. Similarly, respective lobular inflammation grade 0/1/2/3 was 14/151/127/17 patients, and ballooning grade 0/1/2 was 53/168/88 patients. According to fibrosis stage F0, F1, F2, F3, and F4, the number of patients in each stage was 46, 136, 37, 70, and 20, respectively.

**Table 1:** Baseline characteristics of 309 patients with NAFLD in the biopsy-proven cohort

	Median (IQR)/*n* (%)
Age (years)	56 (43–65)
Male	135 (44)
BMI (kg/m^2^)	26.6 (24.1–30.1)
DM	119 (39)
HT	131 (42)
DL	194 (63)
**Laboratory data**	
Albumin (g/dl)	4.5 (4.3–4.7)
T-bill (mg/dl)	0.9 (0.7–1.2)
AST (U/l)	49 (32–76)
ALT (U/l)	71 (43–117)
γ-GT (U/l)	59 (41–95)
BUN (mg/dl)	13 (11–16)
Cre (mg/dl)	0.68 (0.57–0.82)
TC (mg/dl)	205 (179–232)
TG (mg/dl)	127 (94–163)
LDL-C (mg/dl)	126 (106–147)
HDL-C (mg/dl)	51 (44–59)
Plt (×10^4^/μl)	21.7 (17.6–26.5)
HbA1c (%)	5.7 (5.4–6.2)
FBG (mg/dl)	108 (97–120)
IRI (mU/l)	12.6 (8.5–19.1)
HOMA-IR	3.5 (2.2–5.5)
Fe (μg/dl)	113 (92–139)
Ferritin (ng/ml)	169 (86–295)
AFP (ng/ml)	3.5 (2.4–5.3)
ATX (mg/l)	0.87 (0.68–1.21)
**Histological findings**	
Steatosis (1/2/3)	96/138/75
Lobular inflammation (0/1/2/3)	14/151/127/17
Ballooning (0/1/2)	53/168/88
Fibrosis (F0/1/2/3/4)	46/136/37/70/20
Advanced fibrosis (F ≥ 3)	90 (29)
NASH (NAS score ≥ 5)	170 (55)

*AFP* alpha-fetoprotein, *ALT* alanine aminotransferase, *AST* aspartate aminotransferase, *ATX* autotaxin, *BMI* body mass index, *BUN* blood urea nitrogen, *Cre* creatinine, *DL* dyslipidemia, *DM* diabetes mellitus, *F* fibrosis stage, *FBG* fasting blood glucose, *γ-GT* gamma-glutamyltransferase, *HbA1c* hemoglobin A1c, *HDL-C* high density lipoprotein cholesterol, *HOMA-IR* homeostasis model assessment of insulin resistance, *HT* hypertension, *IQR* interquartile range, *IRI* immunoreactive insulin, *LDL-C* low density lipoprotein cholesterol, *NAFLD* non-alcoholic fatty liver disease, *NASH* non-alcoholic steatohepatitis, *Plt* platelet count, *T-bil* total bilirubin, *TC* total cholesterol, *TG* triglycerides.

### Occurrence of events in the biopsy-proven cohort

The median follow-up evaluation period for the 309 patients in the biopsy-proven cohort was 7.0 years (IQR: 3.8–10.1 years). A total of 12 patients (3.9%; 4 male and 8 female) reached the outcome of death. Liver-related death occurred in 7 patients: liver failure in 4 patients, HCC in 2 patients, and ruptured varices in 1 patient. The cause of death in the remaining 5 patients were diverse (breast cancer, heart failure, renal failure, acute myeloid leukemia, and unknown in 1 patient each). Twenty (6.5%) patients newly developed LRE. The 20 patients with liver-related events, including those detected simultaneously, comprised 9 cases of HCC, 2 cases of hepatic encephalopathy, 8 cases of ascites, and 9 cases of esophagogastric varices (Table 2).

**Table 2:** Details of event occurrences in the biopsy-proven cohort

	All (*n* = 309)	Male (*n* = 135)	Female (*n* = 174)
	Median (IQR)/*n* (%)	Median (IQR)/*n* (%)	Median (IQR)/*n* (%)
**Follow-up (years)**	7.0 (3.8–10.1)	6.7 (3.4–9.5)	7.1 (3.8–10.2)
**Events during follow-up**			
Death	12 (3.9)	4 (3.0)	8 (4.6)
LRE^a^	20 (6.5)	7 (5.1)	13 (7.5)
HCC	9 (2.9)	4 (2.9)	5 (2.9)
Hepatic encephalopathy	2 (0.6)	1 (0.7)	1 (0.5)
Ascites	8 (2.5)	1 (0.7)	7 (4.0)
Esophagogastric varices	9 (2.9)	4 (2.9)	5 (2.9)

*HCC* hepatocellular carcinoma, *LRE* liver-related events, *IQR* interquartile range.

LRE included HCC, hepatic encephalopathy, ascites, and esophagogastric varices.

^a^Including 8 cases in which multiple LRE were found simultaneously: 3 cases of HCC + ascites, 1 case of HCC + varices, 1 case of ascites + varices, 1 case of ascites + encephalopathy, 1 case of ascites + encephalopathy + varices,1 case of encephalopathy + varices.

### Comparison of clinicopathologic features between non-LRE and LRE patients in the biopsy-proven cohort

To identify the predictors of LRE, clinicopathological features at the time of biopsy were compared between non-LRE and LRE patients in the biopsy-proven cohort (Table 3). Patients with LRE had significantly higher age (*p* < 0.001), higher prevalences of DM (*p* = 0.012) and HT (*p* < 0.001), higher levels of fasting blood glucose (FBG, *p* = 0.046), insulin (*p* = 0.049), and ATX (*p* < 0.001), lower prevalence of DL (*p* = 0.009), and lower levels of albumin (*p* < 0.001), TC (*p* < 0.001), LDL-C (*p* < 0.001), and platelet count (*p* < 0.001) compared with non-LRE patients. Regarding pathological findings, LRE patients had a lower steatosis score (*p* = 0.003) along with a higher lobular inflammation score (*p* = 0.013) and fibrosis stage (*p* < 0.001) than did non-LRE patients.

**Table 3:** Comparisons of clinicopathological features at time of biopsy between non-LRE and LRE patients in the biopsy-proven cohort

	Non-LRE (*n* = 289)	LRE (*n* = 20)	
	Median (IQR)/*n* (%)	Median (IQR)/*n* (%)	*p* value
Age (years)	55 (42–64)	65 (60–71)	**<0.001**
Male	128 (44)	7 (35)	0.417
BMI (kg/m^2^)	26.5 (24.0–30.3)	27.1 (25.6–28.9)	0.387
DM	106 (37)	13 (65)	**0.012**
HT	115 ((40)	16 (80)	**<0.001**
DL	186 (64)	8 (40)	**0.009**
**Laboratory data**			
Albumin (g/dl)	4.5 (4.3–4.7)	4.2 (3.9–4.3)	**<0.001**
T-bill (mg/dl)	0.9 (0.7–1.2)	1.0 (0.7–1.3)	0.452
AST (U/l)	48 (31–77)	53 (37–61)	0.702
ALT (U/l)	72 (45–118)	42 (34–75)	0.053
γ-GT (U/l)	58 (41–92)	86 (63–180)	0.236
BUN (mg/dl)	13 (11–16)	14 (12–18)	0.077
Cre (mg/dl)	0.68 (0.57–0.82)	0.61 (0.55–0.72)	0.282
TC (mg/dl)	207 (181–233)	174 (157–186)	**<0.001**
TG (mg/dl)	127 (95–163)	124 (88–157)	0.450
LDL-C (mg/dl)	128 (107–149)	97 (81–116)	**<0.001**
HDL-C (mg/dl)	51 (44–59)	49 (44–58)	0.568
Plt (×10^4^/μl)	22.2 (18.1–26.8)	10.9 (9.5–13.6)	**<0.001**
HbA1c (%)	5.7 (5.3–6.2)	6.0 (5.6–6.6)	0.139
FBG (mg/dl)	107 (97–119)	125 (97–147)	**0.046**
IRI (mU/l)	12.1 (8.4–17.9)	24.3 (16.0–29.5)	**0.049**
HOMA-IR	3.4 (2.2–5.0)	6.9 (5.1–9.5)	0.057
Fe (μg/dl)	112 (91–137)	127 (106–159)	0.169
Ferritin (ng/ml)	168 (85–299)	188 (112–236)	0.434
AFP (ng/ml)	3.4 (2.3–4.9)	6.1 (5.0–8.8)	0.627
ATX (mg/l)	0.85 (0.66–1.12)	1.58 (1.04–1.81)	**<0.001**
**Histological findings**			
Steatosis (1/2/3)	84/131/74	12/7/1	**0.003**
Lobular inflammation (0/1/2/3)	14/145/116/14	0/6/11/3	**0.013**
Ballooning (0/1/2)	53/156/80	0/12/8	0.053
Fibrosis (F0/1/2/3/4)	46/136/34/63/10	0/0/3/7/10	**<0.001**
Advanced fibrosis (F ≥ 3)	73 (25.2)	17 (85.0)	**<0.001**
NASH (NAS score ≥ 5)	158 (54.5)	13 (68.4)	0.236

*AFP* alpha-fetoprotein, *ALT* alanine aminotransferase, *AST* aspartate aminotransferase, *ATX* autotaxin, *BMI* body mass index, *BUN* bold urea nitrogen, *Cre* creatinine, *DL* dyslipidemia, *DM* diabetes mellitus, *F* fibrosis score, *FBG* fasting blood glucose, *γ-GT* gamma-glutamyltransferase, *HDL-C* high density lipoprotein cholesterol, *HOMA-IR* homeostasis model assessment of insulin resistance, *HT* hypertension, *IQR* interquartile range, *IRI* immunoreactive insulin, *LDL-C* low density lipoprotein cholesterol, *LRE* liver-related events, *NASH* non-alcoholic steatohepatitis, *Plt* platelet count, *T-bil* total bilirubin, *TC* total cholesterol, *TG* triglycerides.

Bold values indicate statistical significance *p* < 0.05.

### Cumulative event incidence rate in the biopsy-proven cohort

**Fig. 2:**
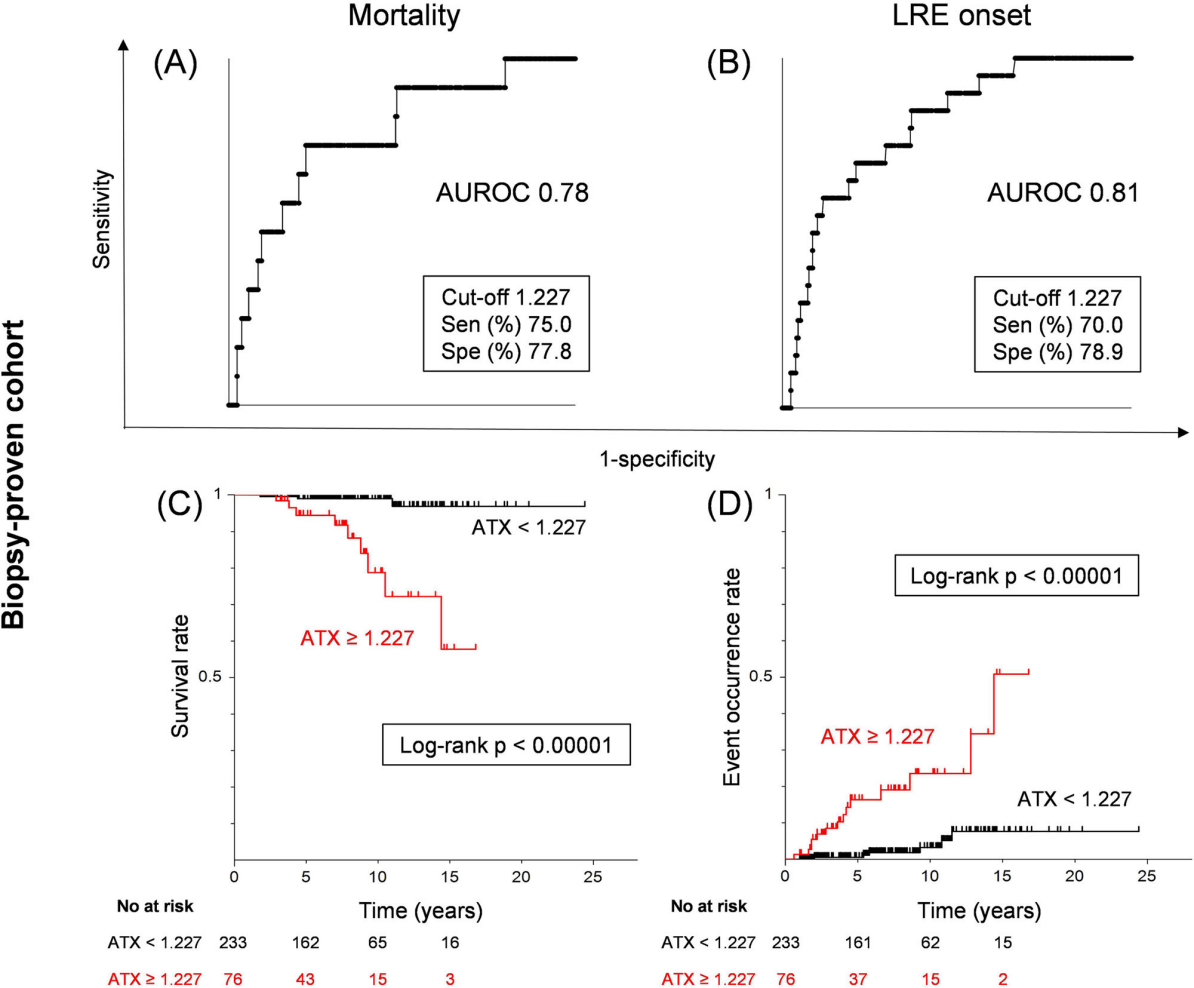
Cumulative event incidence rate analysis of serum ATX levels in the biopsy-proven cohort. Receiver operating characteristic analysis of serum ATX levels for mortality (**A**), and LRE (**B**). Cumulative event incidence rate analysis by the Kaplan–Meier method of serum ATX levels for mortality (**C**), and LRE (**D**). ATX autotaxin, AUROC area under the receiver operating characteristic curve, LRE liver-related events, Sen sensitivity, Spe specificity.

Based on the receiver operating characteristic analysis, we determined serum ATX cut-off values for the outcomes of mortality, and LRE in the biopsy-proven cohort (Fig. 2A, B). The AUROC for predicting death was 0.78. The cut-off value for predicting death was 1.227 mg/l, with corresponding values for sensitivity, and specificity of 75.0%, and 77.8%, respectively. Similarly, for the prediction of LRE, the AUROC was as high as 0.81. The cut-off value was 1.227 mg/l, with corresponding values for sensitivity, and specificity of 70.0%, and 78.9%, respectively.

Chi-square testing was performed to compare incidence rates of low-ATX and high-ATX groups as defined by cut-off values. The incidence rates for death, and LRE were all higher in the high-ATX group (11.8% vs. 1.3%; *p* = 0.00014, and 18.4% vs. 2.7%; *p* < 0.00001, respectively).

Kaplan–Meier survival analysis using the cut-off value of 1.227 mg/l revealed a significantly lower survival rate in high-ATX patients than in low-ATX patients (log-rank *p* < 0.00001). The cumulative LRE incidence rates were higher in high-ATX patients (log-rank *p* < 0.00001) (Fig. 2C, D). The above results indicated that serum ATX levels at the time of biopsy could be a useful parameter for predicting future death and LRE.

### Cumulative event incidence rate by gender in the biopsy-proven cohort

**Fig. 3:**
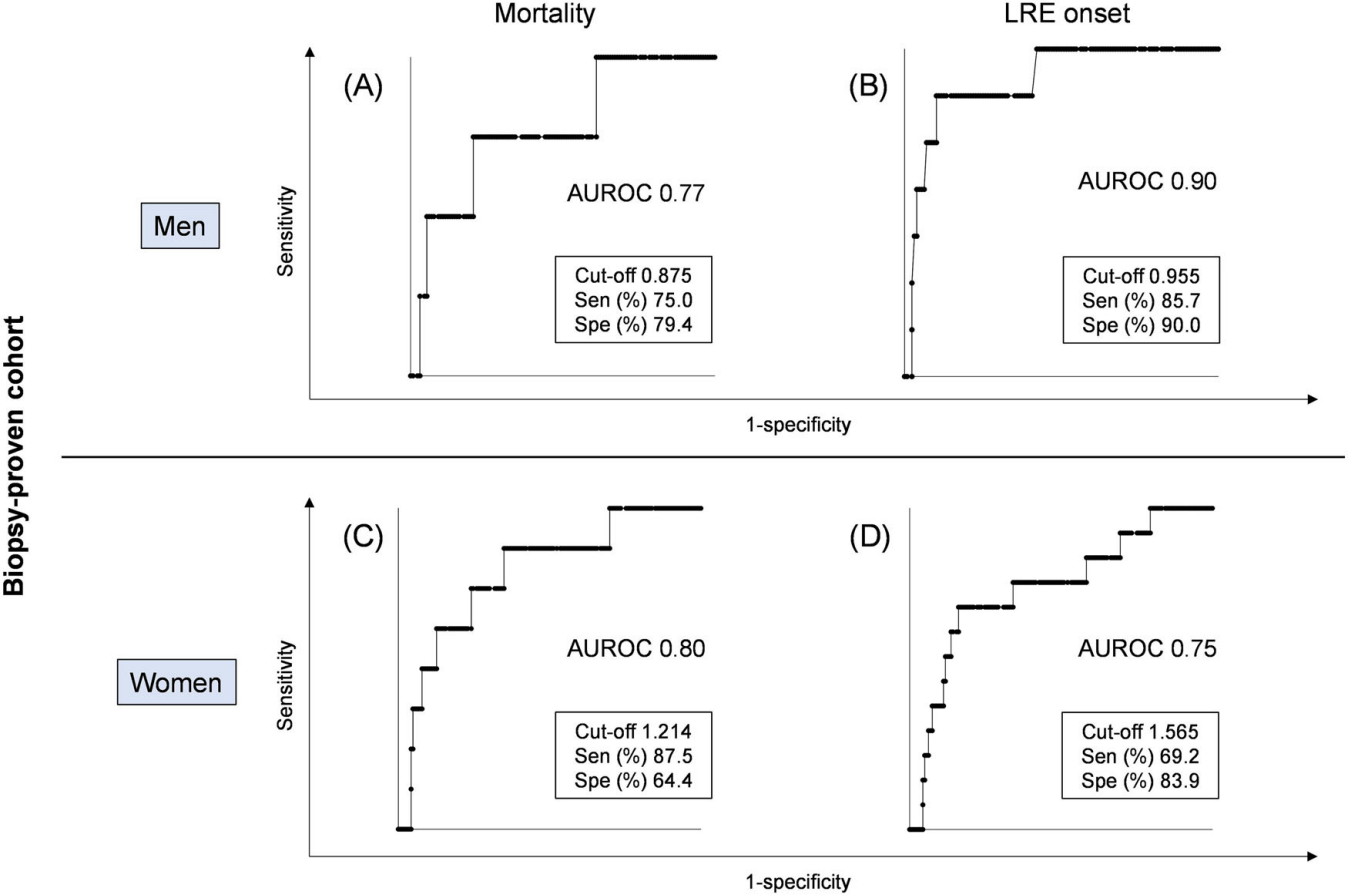
Receiver operating characteristic analysis of serum ATX levels for event occurrence by gender in the biopsy-proven cohort. Receiver operating characteristic analysis of ATX levels for mortality (**A**), and LRE (**B**) in men. Receiver operating characteristic analysis of ATX values for mortality (**C**), and LRE (**D**) in women. ATX autotaxin, AUROC area under the receiver operating characteristic curve, LRE liver-related events, Sen sensitivity, Spe specificity.

**Fig. 4:**
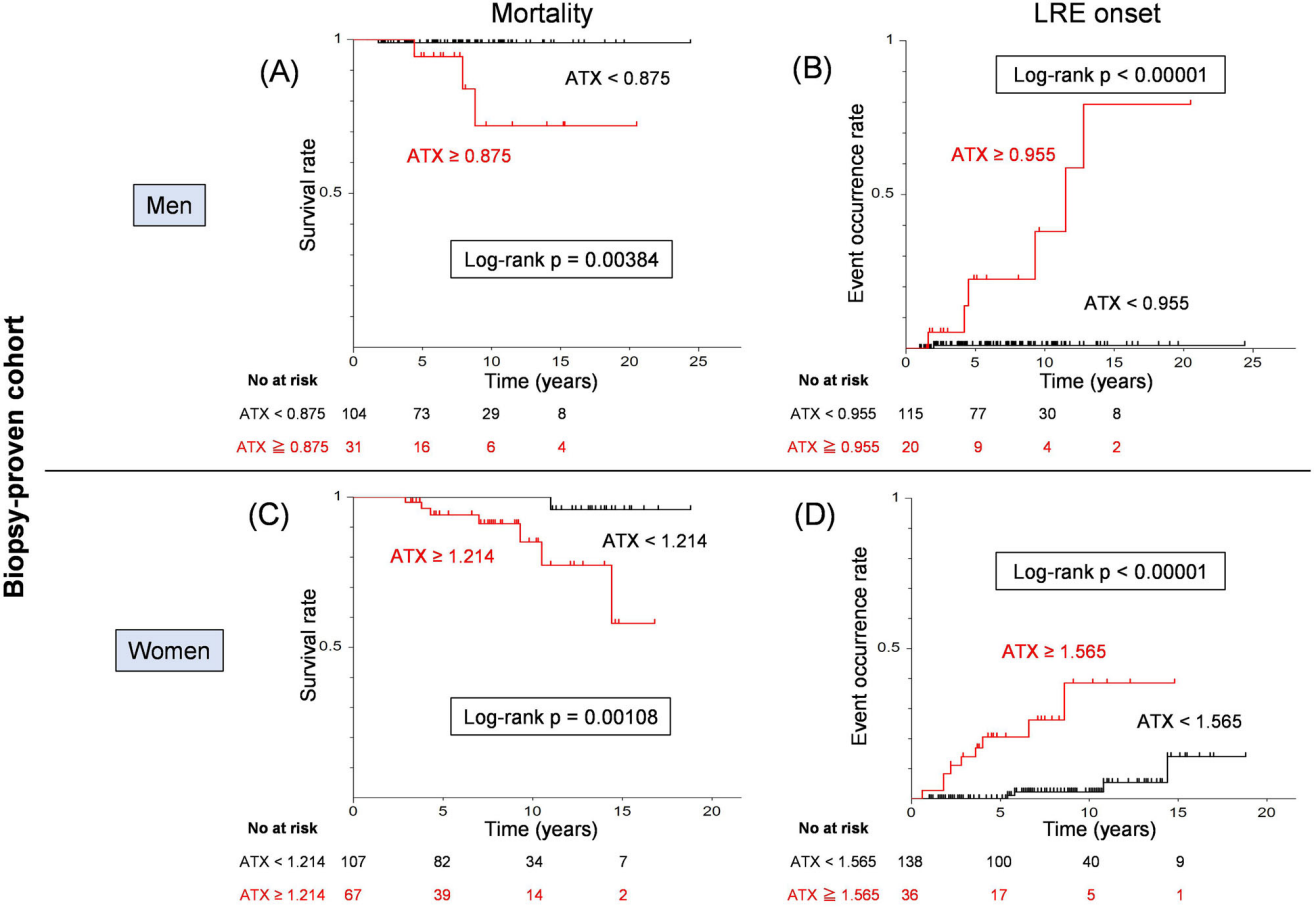
Cumulative event incidence rate analysis of serum ATX levels by gender in the biopsy-proven cohort. Cumulative event incidence rate analysis by the Kaplan–Meier method of serum ATX levels in men for mortality (**A**), and LRE (**B**). Cumulative event incidence rate analysis by the Kaplan–Meier method of serum ATX levels in women for mortality (**C**), and LRE (**D**). ATX autotaxin, AUROC area under the receiver operating characteristic curve, LRE liver-related events.

Previous research has consistently highlighted sex differences in serum ATX levels^29^. In agreement with this, we assessed the utility of serum ATX by gender in the biopsy-proven cohort. The AUROC for predicting death was 0.77 for men (Fig. 3A). The optimal cut-off value of ATX for predicting death was 0.875 mg/l for men (sensitivity: 75.0%, specificity: 79.4%). The AUROC for predicting death was as high as 0.80 for women (Fig. 3C). The optimal cut-off value of ATX for predicting death was 1.214 mg/l for women (sensitivity: 87.5%, specificity: 64.4%). Kaplan–Meier survival analysis using the respective cut-off values for each gender indicated a significantly lower survival rate among the high-ATX subgroups for both sexes (male: log-rank *p* = 0.00384, female: log-rank *p* = 0.00108) (Fig. 4A, C).

As shown in Fig. 3B, the AUROC for LRE prediction was as high as 0.90 for men. The optimal cut-off value for serum ATX predicting the onset of LRE was 0.955 mg/l for men (sensitivity: 85.7%, specificity: 90.0%). The AUROC for prediction of LRE was 0.75 for women (Fig. 3D). The optimal cut-off value for serum ATX predicting the onset of LRE was 1.565 mg/l for women (sensitivity: 69.2%, specificity: 83.9%). Kaplan–Meier survival analysis using the respective cut-off values for men and women showed a significantly higher rate of LRE onset in the high-ATX subgroups for both sexes (male: log-rank *p* < 0.00001, female: log-rank *p* < 0.00001) (Fig. 4B, D).

### Univariate and multivariate Cox proportional hazards models for LRE risk determination in the biopsy-proven cohort

The results of the univariate Cox proportional hazards model for LRE risk determination are shown in Supplementary Table 1. Age (hazard ratio [HR] 1.09, 95% confidence interval [CI] 1.04–1.14, *p* < 0.001), HT (HR 5.93, 95% CI 1.98–17.77, *p* = 0.001), DM (HR 2.63, 95% CI 1.05–6.62, *p* = 0.040), advanced fibrosis (HR 18.71, 95% CI 5.43–64.42, *p* < 0.001), and ATX (HR 2.7, 95% CI 1.78–4.12, *p* < 0.001) were associated with LRE, respectively. Subsequently, multivariate Cox proportional hazards models were developed to determine factors associated with LRE (Table 4). Model 1 used age, HT, DM, ATX, and advanced fibrosis as variables based on the results of the univariate analysis and previous clinical findings in NAFLD^4,30,31^. In Model 2, the confounding relationship between ATX and liver fibrosis was investigated using ATX and advanced fibrosis as variables. Either model, ATX and advanced fibrosis was extracted as an independent factor associated with LRE (Model 1: ATX, HR 2.28, 95% CI 1.00–5.20, *p* = 0.049, advanced fibrosis, HR 8.06, 1, 95% CI 2.11–30.70, *p* = 0.002, Model 2: ATX, HR 2.09, 95% CI 1.10–3.96, *p* = 0.024, advanced fibrosis, HR 13.59, 95% CI 3.83–48.22, *p* < 0.001). The concordance index is 0.909 for model 1 and 0.898 for model 2, both of which are notably high.

**Table 4:** Multivariate Cox proportional hazards analysis for LRE in the biopsy-proven cohort

	Multivariate model 1			Multivariate model 2		
	HR	95% CI	*p* value	HR	95% CI	*p* value
Age	1.03	0.98–1.08	0.302	–	–	–
HT	2.57	0.75–8.83	0.134	–	–	–
DM	1.40	0.52–3.74	0.505	–	–	–
ATX	2.28	1.00–5.20	**0.049**	2.09	1.10–3.96	**0.024**
Advanced fibrosis (F ≥ 3)	8.06	2.11–30.70	**0.002**	13.59	3.83–48.22	**<0.001**

*ATX* autotaxin, *CI* confidence interval, *DM* diabetes mellitus, *HR* hazard ratio, *HT* hypertension, *LRE* liver-related events.

Bold values indicate statistical significance *p* < 0.05.

Furthermore, in a competing risk analysis that considered non-liver-related death as a competing event, ATX (HR 2.29, 95% CI 1.22–4.30, *p* = 0.010) was identified as an independent factor associated with LRE, along with advanced fibrosis (HR 8.01, 95% CI 2.10–30.60, *p* = 0.002) (Table 5).

**Table 5:** Competing risk analysis for LRE in the biopsy-proven cohort

	HR	95% CI	*p* value
Age	1.02	0.97–1.08	0.420
HT	2.67	0.75–9.43	0.130
DM	1.33	0.47–3.80	0.590
ATX	2.29	1.22–4.30	**0.010**
Advanced fibrosis (F ≥ 3)	8.01	2.10–30.60	**0.002**

*ATX* autotaxin, *CI* confidence interval, *Coef* coefficient, *DM* diabetes mellitus, *HR* hazard ratio, *HT* hypertension, *LRE* liver-related events.

Bold values indicate statistical significance *p* < 0.05.

### Validation cohort results

The validation cohort consisted of 88 patients diagnosed with NAFLD by ultrasonography. The clinicopathological features of these patients are presented in Supplementary Table 2. Median age at the time of ATX measurement was 52 years, and 41 patients (47%) were male. The elevated complication rates of DM, HT, and DL were 26%, 27%, and 74%, respectively. Median serum ATX was 0.83 mg/l.

**Fig. 5:**
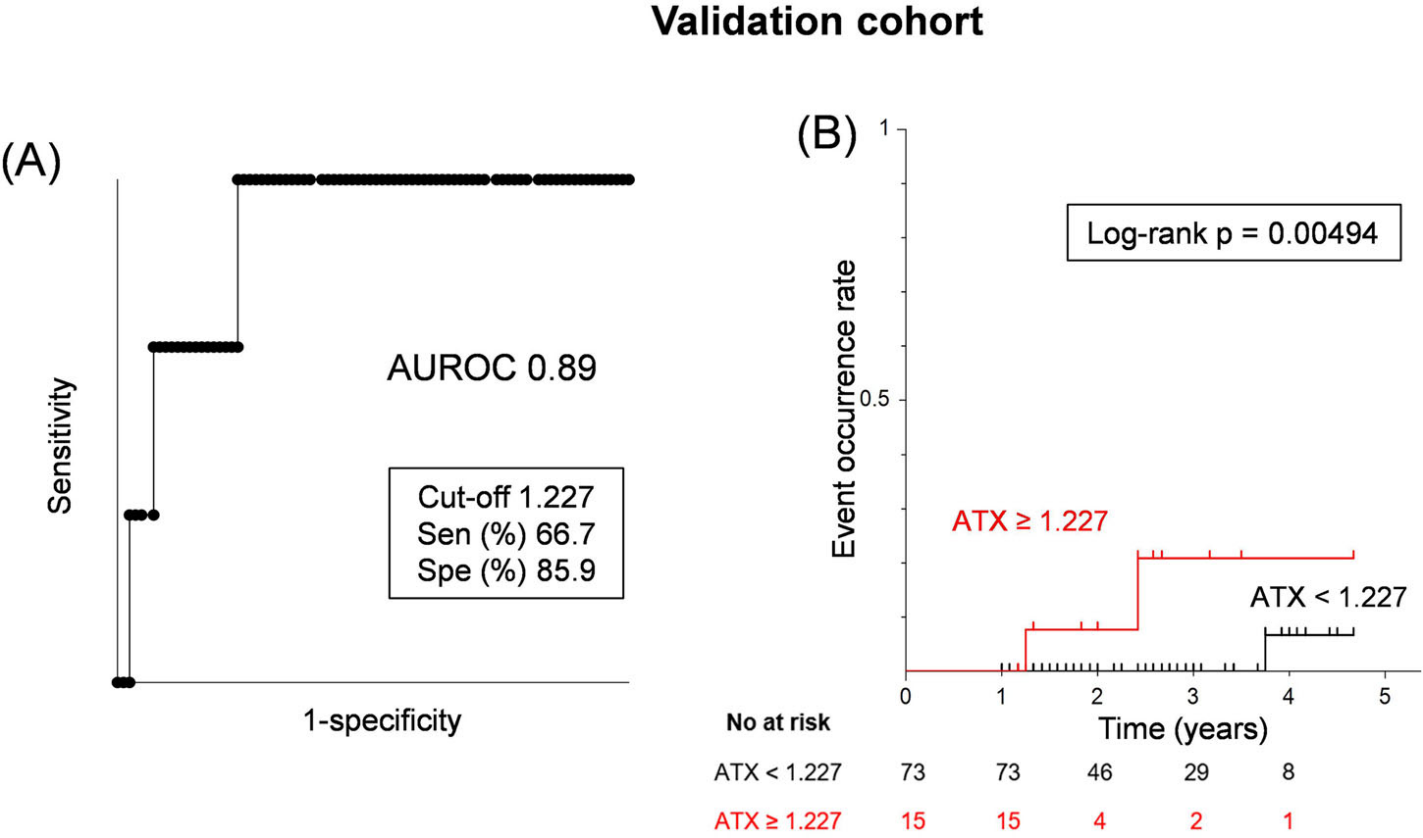
Cumulative LRE incidence rate analysis in the validation cohort. **A** Receiver operating characteristic analysis of ATX for LRE. **B** Cumulative LRE incidence rate analysis by the Kaplan–Meier method of ATX. ATX autotaxin, AUROC area under the receiver operating characteristic curve, LRE liver-related events, Sen sensitivity, Spe specificity.

The median follow-up evaluation period in the validation cohort was 2.5 years (IQR: 1.4–3.4 years) (Supplementary Table 3). LRE was observed in three cases, comprising two instances of HCC and one case of encephalopathy. The clinical features of LRE and non-LRE patients in the validation cohort at the time of ATX measurement are described in Supplementary Table 4. The AUROC for ATX at LRE was very high at 0.89 (Fig. 5A). The LRE screening performance using the cutoff value of ATX 1.227 mg/l in the biopsy-proven cohort (Fig. 2B, D) was a sensitivity of 66.7% and a specificity of 85.9% (Fig. 5A). Kaplan–Meier survival analysis using this cut-off values showed a significantly higher rate of LRE onset in the high-ATX subgroups (log-rank *p* = 0.00494) (Fig. 5B). The validation cohort also indicates the utility of ATX in predicting the development of LRE.

## Discussion

The present study evaluated serum ATX in patients with biopsy-proven NAFLD to determine its potential to estimate disease prognosis, including mortality and the development of HCC and LRE. Receiver operating characteristic analysis revealed ATX cut-off values of 1.227 mg/l for predicting death, and LRE, with corresponding AUROC values of 0.78, and 0.81. Log-rank testing showed high cumulative mortality rate (*p* < 0.00001), and LRE incidence rate (*p* < 0.00001) for ATX cut-off values of 1.227 mg/l. In a multivariate Cox proportional hazards model, it was an unexpected finding that ATX was determined as an LRE-associated factor independent of fibrosis. In addition, an independent validation cohort confirmed the good LRE predictive reproducibility of ATX. Thus, serum ATX exhibited potential utility in predicting such prognostic factors as death, and LRE in patients with NAFLD. To our knowledge, this is the first report evaluating serum ATX and prognosis in NAFLD. Furthermore, in the biopsy-proven NAFLD cohort, 304 out of 308 cases (98.7%) met the criteria for MASLD (all cases of LRE onset were within the MASLD criteria), indicating results that remain applicable even after the change in NAFLD nomenclature.

ATX, a catalyst hydrolyzing LPC to form LPA, has been implicated in liver fibrosis^32^. Specifically, it directly induces fibrosis by stimulating proliferation and contraction of hepatic stellate cells^33^. In fibrotic liver tissue, capillarization of sinusoids impedes ATX uptake, increasing its plasma concentration^34^. Additionally, hepatocyte-secreted ATX exacerbates NAFLD by inhibiting the peroxisome proliferator-activated receptor-α/fibroblast growth factor-21 axis^35^. Clinical studies underscore a significant correlation between serum ATX levels and inflammation/fibrosis in NAFLD^12,36^. This study initially anticipated ATX as a fibrosis biomarker predicting LRE in line with the ATX-fibrosis hypothesis. However, the unexpected and noteworthy finding reveals ATX association with LRE independently of liver fibrosis.

Although further research is essential to elucidate the complex mechanisms of the ATX-LRE relationship, the following discussion explores the possibility that ATX is associated with LRE, apart from the ATX-liver fibrosis hypothesis. First, ATX binds to vascular endothelial growth factor receptors-2 and -3, playing a role in hepatic vessel development and HCC development^32,37,38^. Indeed, ATX is implicated in HCC development not only in chronic viral hepatitis C but also post-antiviral therapy^14,38^. ATX may bind to adhesion molecules like LPA and integrins, potentially contributing to cancer cell metastasis and HCC progression^39^. Secondly, ATX-LPA signaling exhibits diverse effects, including increased ammonia production, heightened permeability of the blood-brain barrier, and induction of hepatic encephalopathy^40–42^. Thirdly, ATX-LPA signaling is also associated with elevated vascular permeability in the liver, potentially causing fluid leakage into the peritoneal cavity, ascites development, and accelerated neovascularization leading to varices formation^43^. Indeed, clinical reports demonstrate elevated serum ATX levels in cirrhotic patients with ruptured varices, ascites, and encephalopathy^44^. Collectively, ATX-LPA signaling emerges as a potential mechanism involved in LRE, such as hepatic encephalopathy, ascites, and esophagogastric varices, independent of liver fibrosis.

Lastly, previous research supports a notable gender difference in reference levels of ATX, with higher values in women^21^. This discrepancy is thought to be influenced by estrogen, which reportedly regulates ATX production and secretion^45^. As such, gender is an important factor to consider when interpreting ATX levels in patients. To account for these physiological and pathological sex differences in ATX, this study included a prognostic analysis of ATX by gender. The utilization of gender-specific cutoffs yielded more accurate and informative prognostic assessments, as illustrated in Figs. 3 and 4.

This study presented that serum ATX levels can predict the occurrence of LRE in patients with NAFLD. However, this study had several limitations, including a retrospective nature, single-center design, and relatively small sample size. Given that the study population consisted exclusively of Japanese individuals, it will be important to validate our findings in larger cohorts of other ethnicities. Moreover, we did not evaluate the time course of serum ATX levels, which could potentially change over time. To establish the clinical significance of ATX in NAFLD, future research involving larger and more diverse patient populations that monitor the changes in serum ATX levels over time will be necessary.

In conclusion, ATX holds promise as a reliable biomarker for predicting the likelihood of adverse long-term outcomes in NAFLD, including death, and LRE. The addition of ATX as a prognostic biomarker may aid in identifying patients at higher risk of adverse outcomes, allowing for more targeted interventions and better disease management.

### Supplementary information

Supplementary information

Description of Additional Supplementary Files

Supplementary Data 1

Reporting Summary

## Data Availability

Source data for the Figures are available as Supplementary data in Excel. The data are available from the authors upon reasonable request.

## Code availability

All statistical analyses in the present study were performed using StatFlex Ver. 7.0　(Artech Co., Ltd., Osaka, Japan) and R software ver. 4.3.0. Further information on code used in the present study is obtained from the corresponding author on reasonable request.
