# Supplementary material for: Serum autotaxin is a prognostic indicator of liver-related events in patients with non-alcoholic fatty liver disease
Source: Commun Med (Lond). 2024 Apr 16;4:73. doi: 10.1038/s43856-024-00499-7 (PMC11021564; doi:10.1038/s43856-024-00499-7)
Supplement: Supplementary file 1 — Supplementary information [file 43856_2024_499_MOESM1_ESM.pdf]

Supplementary Table 1. Univariate Cox proportional hazards analysis for LRE

	**HR**	**95% CI of HR**	**p-value**
Age	1.09	1.04-1.14	**<0.001**
Male	0.72	0.29-1.80	0.479
BMI≥25	2.05	0.69-6.14	0.192
HT	5.93	1.98-17.77	**0.001**
DM	2.63	1.05-6.62	**0.040**
NASH	1.30	0.52-3.27	0.573
Advanced fibrosis (F≥3)	18.71	5.43-64.42	**<0.001**
**Laboratory data**			
T-bill	1.64	0.60-4.46	0.331
AST	1.00	0.99-1.01	0.978
ALT	0.99	0.98-1.00	0.073
γ-GT	1.00	1.00-1.01	0.123
HbA1c	1.21	0.89-1.63	0.219
AFP	1.01	0.99-1.02	0.525
ATX	2.7	1.78-4.12	**<0.001**

AFP, alpha-fetoprotein; ALT, alanine aminotransferase; AST, aspartate aminotransferase; ATX, autotaxin; DM, diabetes mellitus; γ-GT, gamma-glutamyltransferase; F, fibrosis stage; HT, hypertension; IQR, interquartile range; LRE, liver-related events; NASH, non-alcoholic steatohepatitis; T-bil, total bilirubin; ULN, upper limit of normal.

Supplementary Table 2. Baseline characteristics of the validation cohort (n = 88)

	**Median (IQR) / n (%)**
Age (years)	52 (41-67)
Male	41 (46.6)
DM	23 (26.1)
HT	24 (27.3)
DL	65 (73.9)
**Laboratory data**	
Albumin (g/dL)	4.4 (4.1-4.6)
T-bill (mg/dL)	0.7 (0.5-0.9)
AST (U/L)	38 (25-51)
ALT (U/L)	50 (31-76)
γ-GT (U/L)	48 (34-93)
BUN (mg/dL)	15 (12-18)
Cre (mg/dL)	0.78 (0.67-0.93)
TC (mg/dL)	202 (177-235)
TG (mg/dL)	147 (106-207)
LDL-C (mg/dL)	128 (103-149)
HDL-C (mg/dL)	50 (43-58)
Plt ($\times 10^4/\mu L$)	24.5 (19.2-28.9)
HbA1c (%)	6.0 (5.8-6.4)
FBG (mg/dL)	108 (98-125)
AFP (ng/mL)	2.8 (2.1-4.2)
ATX (mg/L)	0.83 (0.69-1.10)

AFP, alpha-fetoprotein; ALT, alanine aminotransferase; AST, aspartate aminotransferase; ATX, autotaxin; DL, dyslipidemia; DM, diabetes mellitus; FBG, fasting blood glucose; γ-GT, gamma-glutamyltransferase; HDL-C, high density lipoprotein cholesterol; HT, hypertension; IQR, interquartile range; LDL-C, low density lipoprotein cholesterol; NAFLD, non-alcoholic fatty liver disease; Plt, platelet count; T-bil, total bilirubin; TC, total cholesterol; TG, triglycerides.

Supplementary Table 3. Details of event occurrences in the validation cohort

	All (n = 88)	Male (n = 41)	Female (n = 47)
	**Median (IQR) / n (%)**	**Median (IQR) / n (%)**	**Median (IQR) / n (%)**
Follow-up (years)	2.5 (1.4-3.4)	2.4 (1.4-3.1)	2.6 (1.4-3.7)
Events during follow-up			
LRE	3 (3.4)	2 (4.9)	1 (2.1)

LRE, liver-related events; IQR, interquartile range

Supplementary Table 4. Comparisons of clinicopathological features between non-LRE and LRE patients in the validation cohort

	Non-LRE (n = 85)	LRE (n = 3)	
	**Median (IQR) / n (%)**	**Median (IQR) / n (%)**	**p-value**
Age (years)	51 (40-66)	72	**0.025**
Male	39 (46)	2 (67)	0.503
DM	21 (25)	2 (67)	0.104
HT	22 (26)	2 (67)	0.119
DL	65 (77)	0 (0)	**0.003**
**Laboratory data**			
Albumin (g/dL)	4.4 (4.2-4.6)	3.7	**0.014**
T-bill (mg/dL)	0.7 (0.5-0.9)	1.0	0.087
AST (U/L)	38 (25-51)	41	0.483
ALT (U/L)	51 (31-76)	39	0.448
γ-GT (U/L)	47 (34-83)	112	0.295
BUN (mg/dL)	15 (12-18)	19	**0.040**
Cre (mg/dL)	0.77 (0.67-0.92)	1.57	0.171
TC (mg/dL)	203 (181-236)	150	**0.005**
TG (mg/dL)	148 (107-206)	127	0.525
LDL-C (mg/dL)	130 (103-149)	89	**0.011**
HDL-C (mg/dL)	50 (43-58)	37	0.094
Plt ($\times 10^4/\mu L$)	24.8 (19.6-28.9)	10.8	**0.007**
HbA1c (%)	6.0 (5.8-6.3)	6.5	0.688
FBG (mg/dL)	108 (98-125)	123	0.258

AFP (ng/mL)	2.7 (2.1-4.1)	4.2	0.508
ATX (mg/L)	0.82 (0.69-1.04)	1.50	**0.022**

AFP, alpha-fetoprotein; ALT, alanine aminotransferase; AST, aspartate aminotransferase; ATX, autotaxin; BUN, bold urea nitrogen; Cre, creatinine ;DL, dyslipidemia; DM, diabetes mellitus; FBG, fasting blood glucose; γ-GT, gamma-glutamyltransferase; HDL-C, high density lipoprotein cholesterol; HT, hypertension; IQR, interquartile range; LDL-C, low density lipoprotein cholesterol; LRE, liver-related events; Plt, platelet count; T-bil, total bilirubin; TC, total cholesterol; TG, triglycerides.
