# Supplementary material for: Serum autotaxin is a prognostic indicator of liver-related events in patients with non-alcoholic fatty liver disease
Source: Commun Med (Lond). 2024 Apr 16;4:73. doi: 10.1038/s43856-024-00499-7 (PMC11021564; doi:10.1038/s43856-024-00499-7)
Supplement: Supplementary file 2 — Description of Additional Supplementary Files [file 43856_2024_499_MOESM2_ESM.pdf]

Description of Additional Supplementary Files

File name: Supplementary Data 1

Description: Supplemental data for patient information.
